# Early warning of regime switching in a financial time series: A heteroskedastic network model

**DOI:** 10.1371/journal.pone.0333734

**Published:** 2025-10-08

**Authors:** Linxi Wang, Sufang An, Zhiliang Dong, Xiaojuan Dong, Jiapei Li

**Affiliations:** 1 School of Urban Geology and Engineering, Hebei GEO University, Shijiazhuang, China; 2 School of Management, Hebei GEO University, Shijiazhuang, China; 3 Strategy and management base of mineral resources in Hebei Province Hebei GEO University, Shijiazhuang, China; 4 Hebei Key Laboratory of Geotechnical Engineering Safety and Deformation Control, Cangzhou, Hebei, China; Universidade Federal do Rio Grande do Sul, BRAZIL

## Abstract

Regime switching in a time series is an important and challenging issue in complex financial system analysis. Existing regime models have focused on the features of fluctuations at a single point in financial time series, often neglecting time series nonlinearity and uncertainties from a dynamic perspective. This study proposes a heteroskedastic network combined with a Hidden Markov Model, the ARMA-GARCH model, and a machine learning algorithm to characterize the dynamic process of a fluctuation in a time series which can uncover the hidden structure of a nonlinear time series with uncertainty. The network community structure can be used to detect regime switching and its early warning signals. We select the S&P 500 time series as our sample data. Our findings indicate that the critical switches between regimes can be detected across various typical periods, and we analyze them from the perspective of the fundamentals and trader expectations in financial markets. The evolution features of regime switching and its early warning signals are also analyzed over the entire sample period. In particular, the critical features of early warning signals can be extracted. This study not only expands regime switching research in time series analysis but also provides a strong theoretical basis for early warning of risk in financial markets for policy-makers and market investors.

## 1 Introduction

Financial time series are an essential framework for comprehending dynamic processes in complex financial systems, such as stock systems [1,2], exchange systems [3], and energy systems [4]. Regime switching is an important and challenging topic in financial time series analysis, where it indicates that the time series suddenly switches from one type of regime to another [5,6]. Regime detection models have been developed and applied in financial markets for risk monitoring. For example, Xie, Fang [7] established the random forest model to effectively warn of tail risk in the global energy market. Huaqing, Yunan [8] utilized deep learning algorithms to construct models for detecting critical shifts in financial risks to cope with uncertainties and risks in financial markets. Ren, Yang [9] constructed a model for evaluating the operational efficiency of the power market using the cloud entropy optimization algorithm to determine the threshold of the critical shift in the operational efficiency of the power market. The purpose of monitoring and recognizing the critical shift in power market operation efficiency is achieved. Duan, Zhao [10] built the bootstrap aggregating-gray wolf optimizer-support vector machine (bagging-GWO-SVM) model to detect the risk of the commodity housing market. Yuanyuan, Zibo [11] confirmed the effectiveness of high-frequency heterogeneous autoregressive (HAR) models in predicting the volatility of the crude oil market. Additionally, traditional econometric methods, such as linear regression models [12–14] and Markov models [15,16], have also been commonly applied in financial time series forecasting studies. Goswami, Boers [6] constructed a network that represents the recurrence probability of a time-ordered sequence of probability density functions and detected abrupt transitions by analyzing the community structure of the network.

Most existing methods applied to financial time series focus on the features of fluctuations at a single point in financial time series. However, there are some challenges in the regime detection models. (1) If the fluctuations of a time series exhibit only small changes before the regime switching occurs, the typical feature of fluctuations at a single point is difficult to be extracted [17]. (2) The regime of a financial time series is influenced by the typical external events, such as the COVID-19 pandemic and the Russia–Ukraine war [18,19]. This suggests that the fluctuations of a time series become more uncertain. The Granger representation theorem describes the adjustment process from short-term fluctuations among multivariate time series to long-term equilibrium [20]. This finding indicates that the dynamic process of fluctuations in a time series can reveal more hidden structures to describe the nonlinear and uncertain feature of a time series.

Time series complex network [21–23] provides a powerful framework for investigating the dynamic processes of fluctuations in financial time series [24,25]. The main idea is to transform the time series into complex networks based on economic models, and then the topological structure of networks can be analyzed to investigate the dynamic feature of fluctuation of financial time series. For example, Dong, Jia [26] established a combination of a quantitative causality analysis model and a complex network to study the quantitative causality of stock price fluctuations among listed companies in the rare earth industry and identified the main fluctuation paths among companies in each segment. Wang, Zhao [27] used a combination of artificial intelligence algorithms and complex networks to predict the volatility of oil time series. Jia, Dong [28] integrated complex network theory and the sliding window method to study the evolution characteristics of short-term causality between crude oil, gold, and the US dollar over a 60-day period from a dynamic perspective. Zhou, Sun [29] built a covariation threshold network to obtain the covariation characteristics between crude oil inventories and the US dollar. Zhen, Tian [30] constructed a complex network based on multidimensional temporal data with the aim of studying the correlation between variables and their dynamic evolution process.

This paper proposes a heteroskedasticity network for early warning of regime switching in financial time series based on a Hidden Markov Model, the ARMA-GARCH model [31–34], and a machine learning algorithm. Our proposed model can detect the regime switching and its early warning signals in an univariable financial time series. This paper offers at least two contributions. First, we map a financial time series into a heteroskedasticity network, which characterizes the dynamic process of fluctuation in a time series with nonlinearity and uncertainty. The reconstructed network can reveal significantly more hidden structures, indicating that our proposed model can provide more information than the fluctuation of a single point in a time series. Second, the network community structure can help to detect the regime switching and its early warning signals during different typical periods. The evolutionary and typical features of regime switching and its early warning signals can be extracted. The S&P 500 time series can be used to test the model. This study not only expands regime switching research in complex systems but also provides a strong theoretical basis for risk management in financial markets for policymakers and market investors.

The rest of the paper is organized as follows. Section 2 presents the data and methodology. Section 3 reports the results of the empirical analysis of the S&P 500. Section 4 discusses the conclusions.

## 2 Methodology and measurement

**Fig 1:**
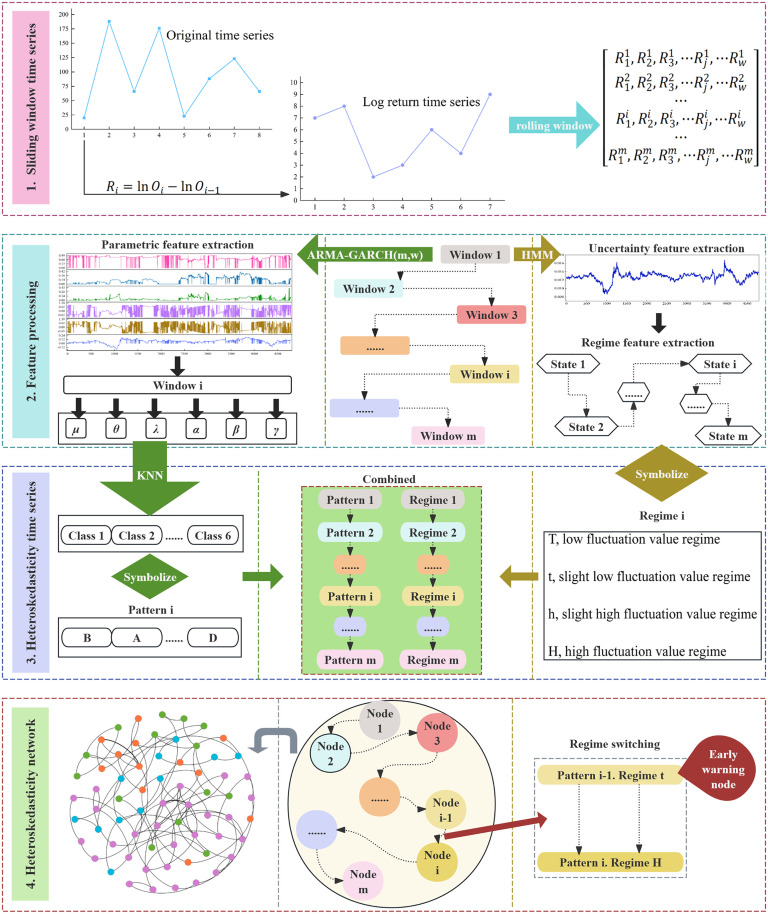
A sample model illustrating the process.

This section describes the methodology and process of studying financial time series. Fig 1 portrays the line of research in this paper. The entire research process is divided into four parts. First, the logarithmic yield time series is obtained from the original financial time series. The sliding window method cuts the logarithmic yield time series. Second, the parametric features of the time series under each window are extracted using the ARMA-GARCH model. The uncertainty and classification features of each time series are extracted using Hidden Markov Model. Third, the parametric and categorical features under each window are symbolized. The data are combined in chronological order. Heteroskedasticity time series are generated for this purpose. Fourth, the heteroskedasticity time series is mapped into a network.

### 2.1 Methodology

In this paper, an early warning node model based on a heteroskedasticity network is proposed. The whole process of the model is shown in Fig 1. First, the entire financial time series is subjected to the sliding window approach. The entire financial time series is then divided into many financial time series segments. Second, the degree of uncertainty of each financial time series segment under each window is calculated. The Hidden Markov Model is then applied to each uncertainty to calculate the regimes belonging to each uncertainty. The heteroskedasticity parameter for each financial time series segment is calculated using the ARMA-GARCH model. Third, to calculate the category of each heteroskedasticity parameter, the heteroskedasticity parameter was applied using the KNN algorithm. Regimes and categories are symbolized. Each window’s categories are symbolized to generate a pattern. The regimes under each window and the pattern were combined to form the heteroskedasticity time series. Fourth, a complex network is constructed based on the transformation between regimes. The patterns of regime transitions are examined based on the network’s topological features and each regime’s heteroskedasticity parameters. Volatility alerts for the financial variable can be identified.

#### Step 1: Sliding window.

We compute the logarithmic rate of return on the raw financial data. The sliding window methodology is then used to generate distinct financial time series segments from the log return series. We obtained the log return of the financial time series as follows, where. $O_{i}$ is the financial variable on day i and $R_{i}$ is the log return of the financial variable on day i. As is followed:


$$R_{i}=\text{In }O_{i}-\text{In }O_{i-\text{1}},\;$$ (1)

The log return series is then divided into segments by applying the sliding window method, where w is the length of the window, $\left\{R_{i},R_{i+\text{1}},\cdots R_{i+w-\text{1}}\right\}$ represents the data of the i-th window, and ${\left\{W_{i}\right\}}_{i=\text{1}}^{m}$ has a certain amount of memory and transmission. The fluctuations in different segments are characterized differently.


$$\left[\;\begin{array}{c} R_{\text{1}},R_{\text{2}},\cdots R_{w}\\ R_{\text{2}},R_{\text{3}},\cdots R_{w+\text{1}}\\ \vdots \\ R_{i},R_{i+\text{1}},\cdots R_{i+w-\text{1}}\\ \vdots \\ R_{m},R_{m+\text{1}},\cdots R_{n} \end{array}\;\right]=\left[\begin{array}{c} \;R_{\text{1}}^{\text{1}},R_{\text{2}}^{\text{1}},R_{\text{3}}^{\text{1}},{\cdots R}_{j}^{\text{1}},\cdots R_{w}^{\text{1}}\;\\ R_{\text{1}}^{\text{2}},R_{\text{2}}^{\text{2}},R_{\text{3}}^{\text{2}},{\cdots R}_{j}^{\text{2}},\cdots R_{w}^{\text{2}}\\ \cdots \\ R_{\text{1}}^{i},R_{\text{2}}^{i},R_{\text{3}}^{i},{\cdots R}_{j}^{i},\cdots R_{w}^{i}\\ \cdots \\ R_{\text{1}}^{m},R_{\text{2}}^{m},R_{\text{3}}^{m},{\cdots R}_{j}^{m},\cdots R_{w}^{m} \end{array}\right]=\left[\;\begin{array}{c} {\begin{array}{c} W \end{array}}_{\text{1}}\\ W_{\text{2}}\\ \cdots \\ W_{i}\\ \cdots \\ W_{m} \end{array}\right],$$ (2)

#### Step 2: Feature processing.

Step 2.1: Regime feature extraction. After the uncertainty of these windows is measured, the regime corresponding to each uncertainty is calculated using the Hidden Markov Model. Let $W_{i}^{avg}$ stand for the average value of window $W_{i}$. We obtain a sequence ${\left\{W_{i}^{avg}\right\}}_{i=\text{1}}^{m}$ from the sequence of windows ${\left\{W_{i}\right\}}_{i=\text{1}}^{m}$. Based on the average value of each window, the uncertainty $W_{i}^{aff}$ under each window is calculated using a machine learning algorithm. From the sequence of windows ${\left\{W_{i}\right\}}_{i=\text{1}}^{m}$, we obtain a sequence ${\left\{W_{i}^{aff}\right\}}_{i=\text{1}}^{m}$. The equation for calculating the uncertainty $W_{i}^{aff}$ under each window is as follows [35]:


$$W_{i}^{aff}\left(W_{i}^{avg};\;a,b,c\right)=\frac{\text{1}}{\left[\text{1}+{\left|\frac{\left(W_{i}^{avg}-c\right)}{a}\right|}^{\text{2}b}\right]},$$ (3)

where a is the width of the uncertainty function (the larger the value, the wider the function); b is the steepness of the uncertainty function (the larger the value, the steeper the function); c is the center position of the uncertainty function (the point where the membership degree is 1).

The Hidden Markov Model (HMM) solves the most likely sequence of states based on the existing observed sequence. The Hidden Markov Model originated from the development of Markov chains because the complexity of practical problems usually exceeds the scope that Markov chains can cover. The uncertainty time series ${\left\{W_{i}^{aff}\right\}}_{i=\text{1}}^{m}$ is subjected to the Hidden Markov Model in this paper. Uncertainty does not always correspond directly to a particular regime. The regime cannot be observed. The regime can only be obtained through data learning and mining on partial uncertainty. There are potential switches between each regime. By calculating the probability of regime switching, the regimes of other parts can also be predicted. This is fundamental to the Hidden Markov Model, which is used to address the problem of uncertainty prediction.

The Hidden Markov Model summarizes three main problems, namely, assessment, decoding, and learning. The evaluation problem calculates the probability of the occurrence of the observation sequence ${\left\{W_{i}^{aff}\right\}}_{i=\text{1}}^{m}$ when the model parameter τ is known and when $F_{i}$ is the regime sequence. The formula is as follows:


$$A(W_{i}^{aff}|\tau )=\sum_{F_{i}}A(W_{i}^{aff}|F_{i},\;\tau )*A(W_{i}^{aff}|\tau ),$$ (4)

The goal of the decoding problem is to find the most likely regime order $F_{i}$ given model parameters and observation order ${\left\{W_{i}^{aff}\right\}}_{i=\text{1}}^{m}$, as follows:


$$arg\;{max}_{{\left\{W_{i}^{aff}\right\}}_{i=\text{1}}^{m}}A(W_{i}^{aff}|F_{i},\;\tau ),$$ (5)

The learning problem is to estimate the parameter τ of the model, given the observation sequence ${\left\{W_{i}^{aff}\right\}}_{i=\text{1}}^{m}$, so that the probability of the observation sequence ${\left\{W_{i}^{aff}\right\}}_{i=\text{1}}^{m}$ in the model reaches the maximum, as follows:


$$arg\;{max}_{\tau }A(W_{i}^{aff}|\tau ),$$ (6)

Step 2.2: Parametric feature extraction. The parametric time series corresponding to each financial time series segment was calculated using the ARMA-GARCH model. The yield and fluctuation of financial assets are direct factors that impact investors’ return on income, and phenomena such as sharp peaks and thick tails, fluctuation aggregation, and leverage effects exhibited in the return series pose challenges. The commonly used description method is the ARMA-GARCH (m,w) model. The ARMA-GARCH (m,w) model reflects both the process of mean change of the series and the stochastic process of residual change. The general ARMA (m,w) model for the time series in $W_{i}$ can be denoted as follows:


$$R_{j}^{i}=\mu_{i}+\sum {\mathrm{\backslash nolimits}}_{i=\text{1}}^{m}\theta_{i}R_{j-i}^{i}+a_{j}-\sum {\mathrm{\backslash nolimits}}_{j=\text{1}}^{w}\lambda_{i}a_{j-i},$$ (7)

$R_{j}^{i}$ indicates the yield over several days. Both m and w are nonnegative integers, denoting the order of autoregression and the sliding average, respectively. $\mu_{i}$ is a constant. $\theta_{i}$ is the autoregressive coefficient of the model. $\lambda_{i}$ is the coefficient of the moving average term of the model. $a_{j-i}$ represents the randomized perturbation term with lag period i.

$a_{j}$ can be defined as follows:


$$a_{j}=\sigma_{j}\varepsilon_{j},$$ (8)

Eq. (6) is the general form of the GARCH (m,w) model.


$${\left(\sigma_{j}^{i}\right)}^{\text{2}}=\alpha_{i}+\sum {\mathrm{\backslash nolimits}}_{i=\text{1}}^{m}\beta_{i}{\left(a_{j-\text{1}}^{i}\right)}^{\text{2}}+\sum {\mathrm{\backslash nolimits}}_{j=\text{1}}^{w}\gamma_{i}{\left(a_{j-\text{1}}^{i}\right)}^{\text{2}},$$ (9)

$\alpha_{i}>\text{0},\;\beta_{i}\geq \text{0},\;\gamma_{i}\geq \text{0},\;\sum_{i=\text{1}}^{max(m,w)}\left(\beta_{i}+\gamma_{i}\right)<\text{1}$. Restraints on $\beta_{i}+\gamma_{i}$ guarantee that the unconditional variance of $\beta_{i}$ is finite, whereas its conditional variance ${\left(\sigma_{j}^{i}\right)}^{\text{2}}$ is time dependent. We use the ARMA-GARCH (1,1) model for segment $W_{i}$. A group of heteroskedasticity parameters $\left\{\mu_{i},\;\theta_{i},\;\lambda_{i},\;\alpha_{i},{\;\beta }_{i},\;\gamma_{i}\right\}$ is obtainable.

#### Step 3: Heteroskedasticity time series.

The heteroskedasticity parameters calculated in the previous section for each sliding window are clustered using the KNN algorithm. The categories corresponding to the different parameters for each sliding window are obtained. When using the KNN algorithm for parameter classification, we use Euclidean distance as the distance metric. For two n-dimensional vectors $x=\left(x_{\text{1}},x_{\text{2}},\cdots ,x_{n}\right)$ and $y=\left(y_{\text{1}},y_{\text{2}},\cdots ,y_{n}\right)$, the Euclidean distance is calculated using the following formula [36]:


$$d(x,y)=\sqrt{\sum {\mathrm{\backslash nolimits}}_{i=\text{1}}^{n}{\left(x_{i}-y_{i}\right)}^{\text{2}}},$$ (10)

In order to correspond to the volatility state of the financial sequence, we used the HMM model to divide the volatility state of the financial sequence into four states [37,38]. When using the KNN algorithm for classification, we set k = 4. We use the KNN algorithm to classify each parameter into four categories: Class 1, Class 2, Class 3, and Class 4. The categories are symbolized to generate the corresponding pattern $W_{i}^{pat}$. Then, A, B, C, and D are used to represent Class 1, Class 2, Class 3, and Class 4, respectively. Since each window generates six parameters, the final pattern will be similar to AABBCD. The process of symbolizing states is similar. We use an HMM model to extract the state of each window: the low fluctuation value regime, slightly low fluctuation value regime, slightly high fluctuation value regime, and high fluctuation value regime. These are then represented by T, t, h, and H, respectively. ${\text{F}}_{\text{i}}$ is the sequence of the regime of ${\text{W}}_{\text{i}}$.


$$F_{i}=F\left(W_{i}\right)=\left\{ \;\;\begin{array}{c} T,\;low\;fluctuation\;value\;regime\\ t,\;slightly\;low\;fluctuation\;value\;regime\\ h,\;slightly\;high\;fluctuation\;value\;regime\\ H,\;high\;fluctuation\;value\;regime \end{array}\right.\;$$ (11)

#### Step 4: Construct a heteroskedasticity network.

Heteroskedasticity time series rely on complex network theory. A heteroskedasticity time series node $P_{i}$ can be obtained by combining the pattern $W_{i}^{pat}$ with its corresponding regime $F\left(W_{i}\right)$. Therefore, we obtain node $P_{i}$ as $\left\{W_{i}^{pat},F\left(W_{i}\right)\right\}$. We take the exchange from one node to the next as an edge. The weight of the edge is the frequency of the exchange between the two nodes. In so doing, we reconstruct a directed weighted evolutionary network. If the pattern under a sliding window corresponds to more than one regime, then we estimate the pattern of this window to be divided into the regime with the highest number. The evolution of heteroskedasticity time series allows the analysis of the evolutionary characteristics of variables under different sliding windows.


$$P_{\text{1}}\;\to \;P_{\text{2}}\;\to \;P_{\text{3}}\;\to \;\cdots \;\to \;P_{i}\;\to \;\cdots \;\to \;P_{m},$$ (12)

In this paper, we reconstruct a heteroskedasticity network of financial time series from heteroskedasticity time series and analyze the dynamic characteristics of financial variables. The node of the network is the regime of each sliding window. The edges are the switches between the regimes of two neighboring days. The direction of the edge points to the regimes of the day after one of these two neighboring days. The weights of the edges are the frequencies of the switches between the regimes of the two days. Because each sliding window has been categorized in this paper before constructing the network, the shift of nodes in the network is affiliated with the process of regimes switching. This network can also be viewed as a network of mutual evolution regimes.

### 2.2 Measurements of data

#### 2.2.1 Evolution probability.

To study the fluctuation preferences of the regime in which financial time series are located, we measure the evolutionary probability of the regime. The important metric used for the measurement is the weight, with the following formula:


$$P_{a\to b}=\frac{\sum_{i\in a,\;j\in b}w_{ij}}{\sum_{a,b\in C}\sum_{i\in a,\;j\in b}w_{ij}},$$ (13)

where $w_{ij}$ denotes the number of times node i in regime a is shifted to node j in regime b. $P_{a\to b}$ is the probability of switching from regime a to regime b. C is the set of four regimes: the low fluctuation value regime, the slightly low fluctuation value regime, the slightly high fluctuation value regime, and the high fluctuation value regime.

#### 2.2.2 Weighted out-degree.

The weighted out-degree $D_{out}(i)$ represents the sum of the number of times a node is shifted into other nodes. The formula for the weighted out-degree $D_{out}(i)$ is as follows [28]:


$$D_{out}(i)=\sum {\mathrm{\backslash nolimits}}_{J=\text{1}}^{n}e_{ij},$$ (14)

where i and j denote nodes. $e_{ij}$ denotes the number of shifts from node i to node j. When a node’s out strength increases, it holds a significant position within the network. This increases the probability that the node is an early warning node.

#### 2.2.3 Betweenness centrality.

The betweenness centrality *B(i)* of node *i* in a heteroskedasticity network can be denoted as follows [39]:


$$B(i)=\frac{\sum_{j}^{N}\sum_{k}^{n}\frac{g_{jk}(i)}{g_{jk}}}{n^{\text{2}}-\text{3}n+\text{2},j\neq k\neq i,j<k,},$$ (15)

where N is the number of nodes, $g_{jk}(i)$ is the number of shortest paths from node j to node k through node i, and $g_{jk}$ is the total number of shortest paths from node j to node k. The greater the median centrality of the warning node is, the greater the median importance of the warning node in the network.

## 3 Results

### 3.1 Data

For this paper, we use the daily index of the S&P 500. The S&P 500, which represents more than half of the worldwide equity market and approximately 80–85% of the total market capitalization of the US equity market, serves as the benchmark. As such, it is recognized as the model for global equity index futures contracts. This study utilizes daily closing price data for the S&P 500 over two decades from 1 January 2004 to –2 September 2024. All the data are from the Wind database.

**Fig 2:**
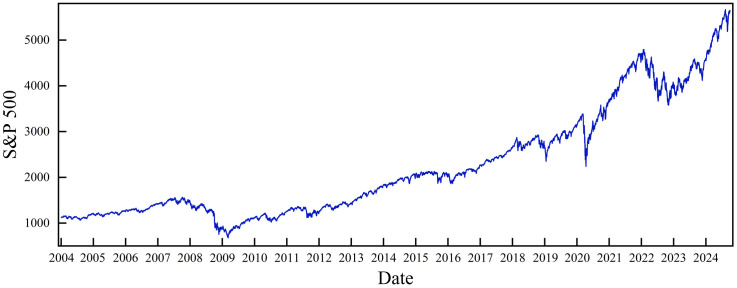
S&P 500 time series.

Fig 2 illustrates the fluctuation of the S&P 500 over a two-decade period. The S&P 500 has exhibited an upward trajectory during this time frame. Notably, between 2007 and 2009, the index experienced its lowest point, likely attributable to the repercussions of the global financial crisis on equity markets. Despite ongoing fluctuations, the S&P 500 subsequently rebounded. However, it faced another sharp decline due to the COVID-19 pandemic from 2019 to 2021 and escalating China‒U.S. trade friction. The 2022–2023 period saw breakout and continuation of the Russia–Ukraine war and intensifying global geopolitical conflict. This is currently the most important risk facing the world economy. Global stock markets have been affected by geopolitical events, and the S&P 500 has fluctuated greatly. Table 1 shows the distribution of the S&P 500 over two decades. To avoid the influence of time changes on the fluctuation of the S&P 500, this paper can use the logarithmic rate of return of the S&P 500 as the research object.

**Table 1:** Descriptive statistics for the S&P 500 time series.

Descriptive statistics	Value
Maximum	5667.20
Minimum	676.53
Average	2241.45
Variance	1445533.92
Standard deviation	1202.30
Median	1878.21

**Fig 3:**
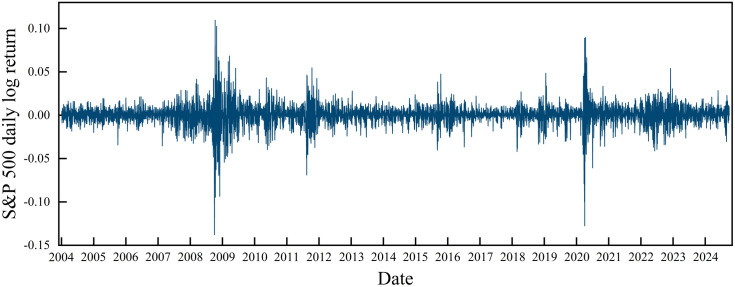
Time series of the daily log returns of the S&P 500.

Based on the steps shown in Section 2, this paper takes the natural log return of the original daily S&P 500. The daily log return data of the S&P 500 are obtained as shown in Fig 3. It fluctuates around the zero value, showing more obvious fluctuation aggregation. The entire log return time series is divided into segments using a sliding window. The fluctuation range of each segment is measured. The window length is chosen to be 240 days of the economic cycle, which is equivalent to one year with weekends and holidays removed [17,24,40,41]. The step size is 1 day.

**Fig 4:**
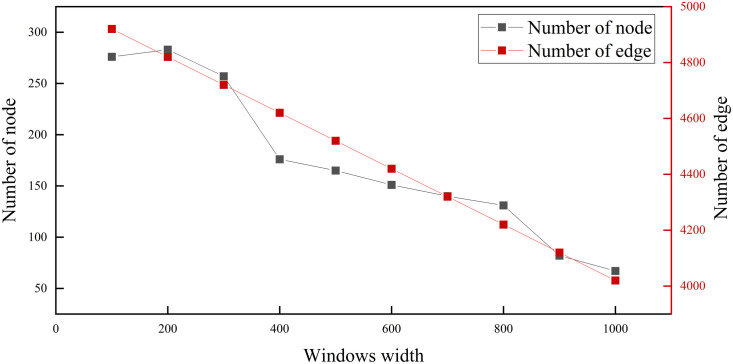
Distribution of nodes and edges in networks with different window lengths.

A sliding window is a fixed-length subsequent of data. Over time, the sliding window slides over the data sequence, processing each subsequent window. In the machine learning method of this paper, a sliding window is used for the time series analysis task. The sliding window allows a long sequence to be decomposed into a series of short sequences, thus facilitating processing and analysis. In short, sliding windows are used for predictive analysis of time series to help decision-makers develop better strategies and plans. Adaptive sliding window sizes and shapes can be designed for different data characteristics and application scenarios to better suit various application requirements. This paper experiments with the effect of combining different window sizes with a complex network approach. In the heteroskedasticity network, the number of network nodes and edges were observed. The heteroskedasticity network complexity is correlated with the quantity of these two measurements. Based on the results of the examination, the size of the window that best suits the object of study of this paper is selected. Fig 4 illustrates how the window size transformation affects the quantity of network nodes and edges. Based on the fluctuation of the curves, we find that the number of nodes and edges in the network decreases as the length of the window increases. For example, Fig 4 shows that at a window width of 100 days, the network has 276 nodes and 4920 edges. At a window width of 1000 days, the network has 67 nodes and 4020 edges. Both the complexity and the diversity of the heteroskedasticity network’s states decrease with increasing window width. Combined with the research object of this paper, this paper chooses the window length as one economic cycle—240 days—which is equivalent to the number of days in a year after removing weekends and holidays [17,24,40,41]. The step length is 1 day. The length of the window has economic significance and presents reasonable network complexity.

### 3.2 Early warning regime switching in different periods

The changes in the S&P 500 can be affected by complex factors. According to previous studies, the characteristics of fluctuations in the S&P 500 can be expressed as fluctuations around a certain average value for a period of time. When the values are near the fundamental-based value, the regime switches. In this paper, we discuss the problem of regime switching based on this factor in conjunction with an established heteroskedasticity network. On the one hand, it is possible to monitor the transitions between the regime switching nodes of one network community and those of another community at various times. On the other hand, it is possible to evaluate the evolutionary traits of regime switching from a broad standpoint.

**Fig 5:**
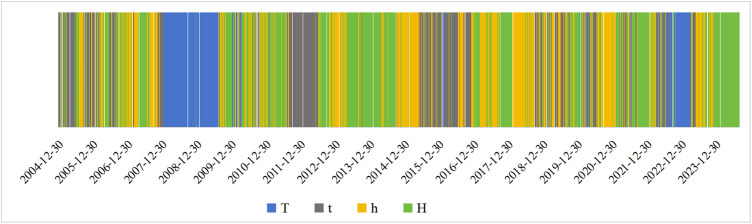
Regime evolution path.

We viewed the temporal distribution of the regime at different times based on the evolution of the regime under different windows. The evolution pattern of the regime can be used to understand how the S&P 500 fluctuates at various points throughout time. The evolution of the regime is shown in Fig 5. In the whole time series, the high fluctuation value regime occurs in the middle and late periods. The low fluctuation value regime mostly occurs in the early and late periods. There is no regularity in the time period in which slightly high and low fluctuation values occur.

**Fig 6:**
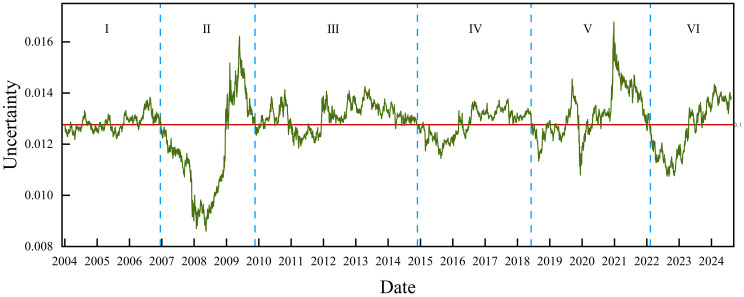
Dynamic uncertainty values of the S&P 500 time series.

Fig 6 shows the time series of the uncertainty of the data in each window. The path of the fold helps us observe the dynamic fluctuations in the logarithmic yield of the S&P 500. The path of the folded line in this picture shows that the uncertainty of the S&P 500 is time-varying. Based on the fluctuation state of the uncertainty time series and typical geopolitical events, we divide the original time period into six periods. Table 2 shows not only the dates corresponding to each stage but also the analysis of the reasons for the fluctuation in the uncertainty in each stage. Based on the transfer of nodes from one regime to another in each period combined with the actual situation, we determine the reasons for the transfer of regimes.

**Table 2:** Time intervals for each phase.

No.	Period	Typical events
Ⅰ	2004.01.05−2007.01.04	Stable economy
Ⅱ	2007.01.05-2009.12.15	Global financial crisis
Ⅲ	2009.12.16-2014.12.15	Economic recovery
Ⅳ	2014.12.16-2018.04.26	Slowdown in world economic growth and economic downturn
Ⅴ	2018.04.27-2022.01.25	COVID-19 pandemic and China‒US trade frictions
Ⅵ	2022.01.26-2024.09.02	Russia‒Ukraine war

In the early part of the first stage, the regimes evolve over time between t and h. This suggests that in the first stage, the S&P 500 gradually overshoots from a slightly low fluctuation value regime to a high fluctuation value regime. In the later part of the first stage, the regimes evolve with time between h and H. During this phase, the global economy is growing steadily. The global stock market is mostly stable and healthy. When the S&P 500 is on the rise, investors become optimistic.

In the second phase, the image mostly shows T. The S&P 500 mostly shows a low fluctuation value regime. This is strongly related to the outbreak of the global financial crisis. Additionally, terrorist attacks frequently occurred around the world, and terrorism constitutes a threat to global security and stability. Markets crashed, and industries were thrown into turmoil. The US dollar continues to weaken. The international prices of gold, crude oil, and agricultural products rose sharply, hitting record highs. The demand for all types of energy dropped sharply, causing dramatic fluctuations in financial markets. Investor confidence was shattered, intensifying turmoil in international financial markets.

The third stage of the regime is a gradual transition from t to H. In this stage, the S&P 500 switches from a low fluctuation value state to a high fluctuation value. This is because the global financial crisis forced countries to focus on their own growth. The long interval in this phase confirms the severity of the global financial crisis in terms of the extent of the damage to the development of each country. Investors regained confidence as the S&P 500 gradually plateaued.

The regime of the fourth stage is mostly characterized by t. The S&P 500 switched from a high fluctuation value regime to a slightly low fluctuation value regime. It then switched again from the slightly low fluctuation value regime to the high fluctuation value regime. The fourth stage of uncertainty was more volatile. It was a dramatic year, with stock markets traveling a circuitous, tumultuous path that eventually returned to its starting point. It declined sharply approximately 2015 and then rebounded. During this period, world industrial production grew at a low rate. Commerce remained slow. Financial market instability worsened, and commodity prices dropped. In developed nations, economic recovery has been sluggish. The rate of growth in emerging economies continued to decline. The overall recovery of the global economy was feeble and gradual. The shocks at this stage have led to negative sentiment among investors.

In the early part of the fifth stage, the regime is dominated by t and h. The S&P 500 transforms between a slightly low fluctuation value regime and a slightly high fluctuation value regime. The fifth stage is a period that represents an economic shock, and although the global economy is growing at a high rate, the S&P 500 still experienced a sharp decline during this stage. This was due to the COVID-19 pandemic, which brought the global economy to a standstill. Major economies, such as the United States, adopted easing policies and introduced fiscal measures for economic stimulus and relief, but the world economy still suffered an unprecedented blow. In the latter part of the fifth stage, the regime switched rapidly from H to T. The S&P 500 switched sharply from the high fluctuation value regime to the low fluctuation value regime. In the second half of 2020, the success of China’s fight against the COVID-19 pandemic and the aggressive process of global vaccine development brought the end in sight for the COVID-19 pandemic. However, the COVID-19 pandemic recurred globally, and in addition to the public health crisis, the impact of the COVID-19 pandemic on poor people, employment, debt problems, and the normal order of life, work, and education continues to linger. By the end of 2020, among the markets of major developed countries and regions around the world, the indices of the United Kingdom, France, and Hong Kong had not yet recovered from the plunge during the COVID-19 pandemic and are still decreasing to varying degrees from the beginning of the year. China‒U.S. trade frictions further dampened the recovery of the world economy. Compared with the beginning of this period, shocks in the world economy brought pessimism to investors.

The sixth stage of regime T continued to appear for some time. The S&P 500 continued in a low fluctuation value range for an extended period of time before rallying. In the past 2022, a combination of forces, including violent Fed rate hikes and the Russia–Ukraine war, continued to impact markets. All three major U.S. stock indices posted their worst annual performance since the 2008 financial crisis. The world economy was hit hard. The global stock indices continued to decline, and the economic situation was not optimistic in an environment where the long-term effects of the COVID-19 pandemic were still being felt. Geopolitical conflicts were intensifying, the international situation was changing in complex ways, and many countries were facing high inflation. This has led to negative sentiment among investors. However, the world economy has a strong foundation, global regional economic cooperation is still strengthening, and cooperation in competition still exists. Trade in the midst of weak global growth has not been completely halted, which makes the continued weak growth trend in 2024 sustainable. Investor sentiment is positive about the future.

### 3.3 Early warning of regime switching over the entire sample period

#### 3.3.1 Evolution probability between regimes.

**Fig 7:**
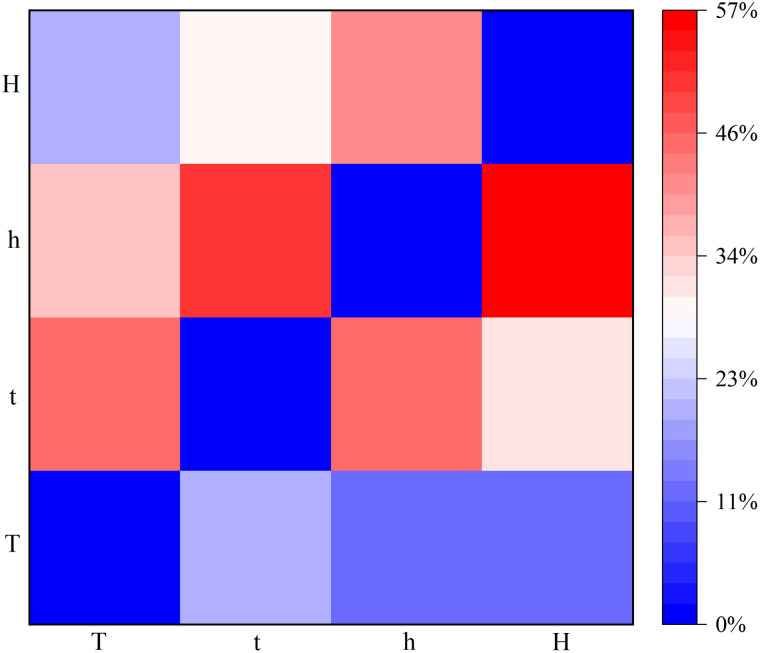
Evolution probabilities between different regimes.

Fig 7 shows the probabilities of different regimes switching to each other. For example, the value of $P_{t\to h}$ is 50.60%. t has a 50.60% probability of evolving to h. This indicates that when the S&P 500 is in a slightly low fluctuation value regime, there is a 50.60% probability of evolving to a slightly high fluctuation value regime. A comparison of the different evolution probabilities of t reveals that the value of $P_{t\to h}$ is twice as high as that of $P_{t\to T}$ or $P_{t\to H}$. This indicates that the slightly high fluctuation value regime is the main evolutionary preference for the slightly low fluctuation value regime. The smallest probability is the evolution probability from a high fluctuation value regime to a low fluctuation value regime, which is 11.95%. This suggests that the probability of large institutional evolutionary preferences occurring is tiny. Each regime will have a different evolutionary preference. When the regime changes, it indicates a large change in the S&P 500. The likely reason for this change is the occurrence of geopolitical events, adjustments in the energy market’s supply and demand, world revolutions, and so on. An overall analysis of the evolutionary preferences of regimes reveals that regimes only evolve to adjacent ranges of regimes. Changes in regimes do not occur in large spanwise jumps. This suggests that the S&P 500 does not rise or fall rapidly but rather increases or decreases in fluctuation. The evolutionary preferences of regimes suggest that the fundamentals and expectations of change are not random.

To further analyze the early warning of critical switches, in this paper, we identify the early warning nodes when critical switches occur. For example, during the switching from a slightly high fluctuation value regime to a slightly low fluctuation value regime, we identify the critical switching nodes for each regime. We subsequently extracted the heteroskedasticity parameters $\left\{\mu_{i},\;\theta_{i},\;\lambda_{i},\;\alpha_{i},{\;\beta }_{i},\;\gamma_{i}\right\}$ of the critical switching nodes. The nodes had significant changes in the heteroskedasticity parameters of the heteroskedasticity time series during the switching: the parameter $\mu_{i}$ was reduced by 5.05%, the parameter $\theta_{i}$ was reduced by 49.81%, and the parameter $\lambda_{i}$ was reduced by 44.81%. These parameters indicate a large difference in the ARMA-GARCH (1, 1) effect of the early warning nodes during the switching from a slightly high fluctuation value regime to a slightly low fluctuation value regime. The impact of multiple factors, such as inflation, the monetary policies of major countries, and geopolitical tensions, can lead to turbulence in global stock markets. In turn, the heteroskedasticity parameter of the S&P 500 changes. Investor sentiment can become strained as these events happen. When the regime changes significantly, investors feel negative. When the S&P 500 rises, it indicates that investors may have an opportunity to benefit from the upward trend in the stock market. Investors can increase their positions through regular investments, such as investing a certain percentage of funds each week. This can help avoid emotional decision-making and mitigate certain risks. The model discussed in this article serves as an early warning mechanism in such situations. Conversely, when the S&P 500 shows a downward trend, investors may consider taking measures to withdraw funds appropriately. In practical applications, the emergence of different types of early warning signals can help investors make more positive decisions.

#### 3.3.2 Evolution probability of early warning signal parameters.

**Fig 8:**
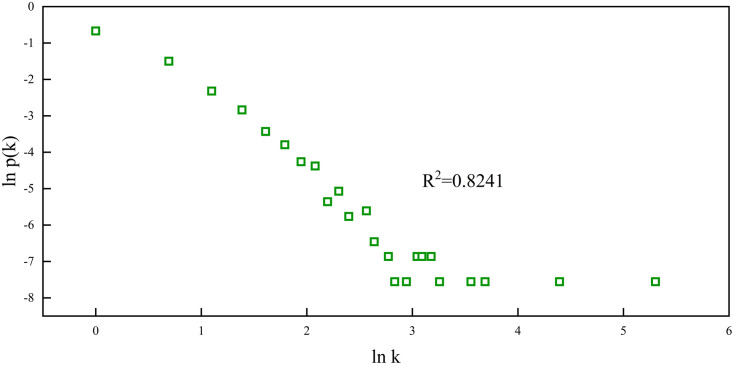
Double logarithmic plot between the weighted out-degree of a node and its probability in a heteroskedasticity network.

The weighted out-degree power-law distribution of nodes in the network is shown in Fig 8, where the distribution of nodes is similar to a long tail. This shape means that most of the individuals have small values, but there will be few individuals with very large values.

**Fig 9:**
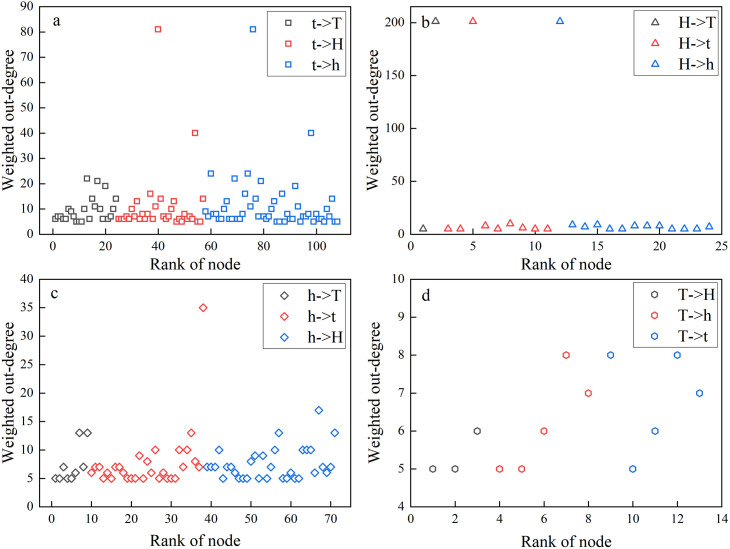
Weighted out-degree of an early warning node in a regime under the following conditions: (a) the critical switching node is in the slightly low fluctuation value regime; (b) the critical switching node is in the high fluctuation value regime; (c) the critical switching node is in the slightly high fluctuation value regime; and (d) the critical switching node is in the low fluctuation value regime.

Only a small number of nodes in this study have a greater probability of switching into other nodes in the regime. The weighted out-degree of nodes follows a power law distribution. This suggests that the main heteroskedasticity characteristics of index fluctuations across several windows are covered by these nodes. As illustrated in Fig 9, we further examine the early warning nodes with high weighted out-degree. For instance, early warning nodes with high output intensities from the slightly low fluctuation value regime to the low fluctuation value regime are represented by the black nodes in Fig 9(a). It is also the corresponding critical switching node in the slightly low fluctuation value regime. The warning nodes with the highest weighted out-degree are the most important evolutionary emitters when the critical switch from the slightly low fluctuation value regime to the low fluctuation value regime occurs.

#### 3.3.3 Evolution probability of betweenness centrality.

Betweenness centrality quantifies how significant a node is as a critical network mediator. In particular, betweenness centrality measures the frequency of critical nodes acting as mediators of all shortest paths in the heteroskedasticity network built in this paper. A node with a higher node betweenness centrality is more likely to be found on the shortest paths between other nodes, giving it comparatively more control and influence over the spread of its effect.

**Fig 10:**
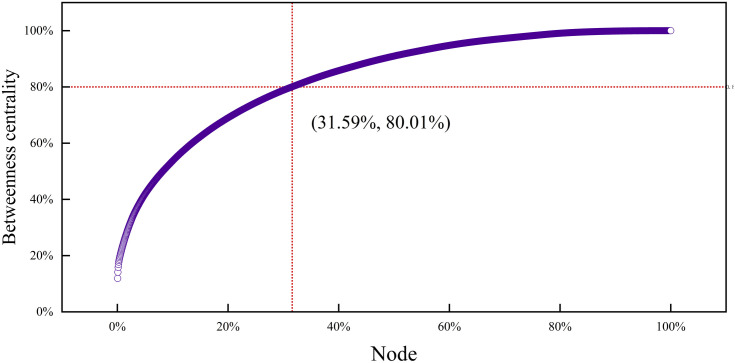
Node betweenness centrality cumulative distribution.

**Fig 11:**
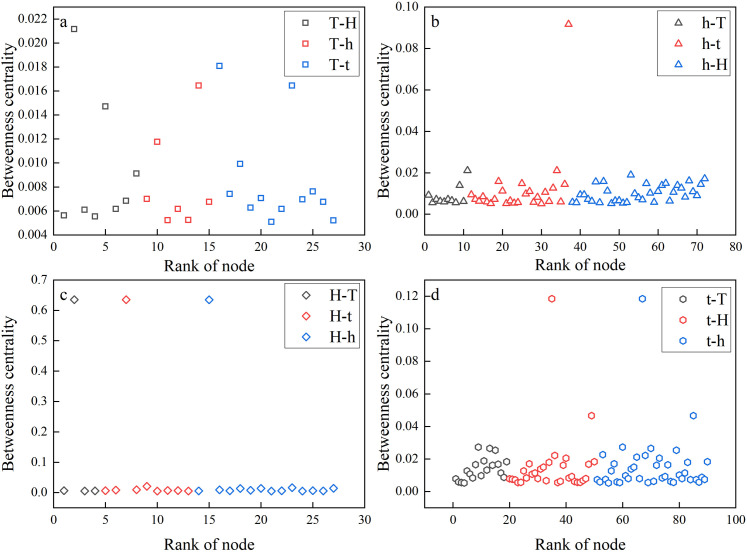
Betweenness centrality of an early warning node in a regime under the following conditions: (a) the critical switching node is in the low fluctuation value regime; (b) the critical switching node is in the slightly high fluctuation value regime; (c) the critical switching node is in the high fluctuation value regime; and (d) the critical switching node is in the slightly low fluctuation value regime.

A few nodes are quite significant in the network and play a critical role in contact switching and information transmission, as illustrated in Fig 10, where 31.59% of the nodes have an overall mediation ability of 80.01%. To understand the significance of early warning nodes based on betweenness in a network, Fig 11 displays the distribution features of the betweenness centrality of early warning nodes. For example, the black nodes in Fig 11(a) are early warning nodes of high betweenness centrality from the low fluctuation value regime to the high fluctuation value regime. It is also the corresponding critical switching node in the low fluctuation value regime. When there is a major transition from a low fluctuation value regime to a high fluctuation value regime, early warning nodes with the highest betweenness centrality have the most significant inter-degree correlation role.

## 4 Conclusions

An early warning node model is proposed in this paper. The model requires the Hidden Markov Model, KNN algorithm, and complex network theory. The study focuses on institutional transition early warning for financial time series. First, we divide the financial time series into segments using a sliding window. Second, we calculate the degree of uncertainty corresponding to each window. Subsequently, the uncertainty is used in the Hidden Markov Model to obtain the regime corresponding to each financial time series segment. We calculated the heteroskedasticity parameters of the financial time series under each window using ARMA-GARCH model. Third, the KNN algorithm was used to obtain the categories corresponding to each set of heteroskedasticity parameters. By symbolizing the regime and the categories, we combine the regimes and patterns corresponding to each window to form a heteroskedasticity time series. Fourth, we use complex network theory to reflect the evolution of the dynamic characteristics of the heteroskedasticity time series. The nodes of the network are the regimes to which each window belongs. We exploit the dynamic properties of heteroskedasticity network time series and complex network dynamics.

The research in this paper provides new evidence for identifying early warning nodes. It can not only help relevant market investors avoid risks but also help policy-makers predict the future movements of relevant markets. Relevant market investors can identify the fluctuation range where financial variables are located, and at the same time, they can also recognize the emergence of early warning nodes. Market investors can profit from or avoid the risk of losing money on early warning nodes that evolve into different fluctuation ranges. Furthermore, market managers can identify possible early warning nodes and trends in index ranges in the event of geopolitical events, particularly whether the state of the economy around the world is stable and political events related to the stock market. This is a tool for maintaining the stability of stock markets.

This study proposes a new early warning model of regime switching in a financial time series based on the ARMA-GARCH model, the KNN algorithm, the HMM, and complex network theory. Although the model performs well, the results are limited by the length of the sliding window, economic models, and datasets. In the future, other volatility models (such as EGARCH or GJR-GARCH) and other machine learning models, such as deep learning framework, can be used to improve the performance of early warning models. We will also explore other dynamical complex network models to expand the applicability of our model.

## Supporting information

S1 Supporting information. (XLSX)
