# Correction: Early warning of regime switching in a financial time series: A heteroskedastic network model

**DOI:** 10.1371/journal.pone.0339698

**Published:** 2025-12-23

**Authors:** Linxi Wang, Sufang An, Zhiliang Dong, Xiaojuan Dong, Jiapei Li

The second and third authors, Sufang An and Zhiliang Dong, should be noted as shared co-corresponding authors on this work. Zhiliang Dong’s email address is: dongzhl@126.com.

## References

[1] Wang L, An S, Dong Z, Dong X, Li J. Early warning of regime switching in a financial time series: A heteroskedastic network model. PLoS One. 2025;20(10):e0333734. doi: 10.1371/journal.pone.0333734 41061022 PMC12507299

